# Recent advances in the elucidation of enzymatic function in natural product biosynthesis

**DOI:** 10.12688/f1000research.7187.2

**Published:** 2016-02-25

**Authors:** Gao-Yi Tan, Zixin Deng, Tiangang Liu

**Affiliations:** 1 Key Laboratory of Combinatorial Biosynthesis and Drug Discovery (Wuhan University), Ministry of Education, and Wuhan University School of Pharmaceutical Sciences, Wuhan, China; 2 Hubei Engineering Laboratory for Synthetic Microbiology, Wuhan Institute of Biotechnology, Wuhan, China

**Keywords:** natural product biosynthesis, plant-derived drugs, combinatorial biosynthesis

## Abstract

With the successful production of artemisinic acid in yeast, the promising potential of synthetic biology for natural product biosynthesis is now being realized. The recent total biosynthesis of opioids in microbes is considered to be another landmark in this field. The importance and significance of enzymes in natural product biosynthetic pathways have been re-emphasized by these advancements. Therefore, the characterization and elucidation of enzymatic function in natural product biosynthesis are undoubtedly fundamental for the development of new drugs and the heterologous biosynthesis of active natural products. Here, discoveries regarding enzymatic function in natural product biosynthesis over the past year are briefly reviewed.

## Introduction

William Campbell, Satoshi Omura, and Tu Youyou jointly won the 2015 Nobel Prize in Medicine or Physiology for discovering the anti-parasite drugs avermectin and artemisinin. Natural products, obtained from bacterial, algal, fungal, plant, and animal sources, have provided humankind with powerful weapons to combat various diseases ^1, 2^. However, many active natural products, especially plant-derived drugs, have several disadvantages, such as low productivity and unstable source supplies. In 2013, artemisinic acid, a pharmaceutical precursor of artemisinin, which is a potent antimalarial drug produced by the sweet wormwood plant *Artemisia annua* ^3^, was first produced in an engineered *Saccharomyces cerevisiae* strain ^4^. Semi-synthetic artemisinin is available on the market, and its production capacity is currently about 50–60 tons per year ^5, 6^. This work marks a milestone in synthetic biology. Very recently, well-known opioids have also been successfully biosynthesized in *S. cerevisiae* ^7^; this is considered to be the most complicated and elaborate engineering work ever achieved in the field, and it is a remarkable landmark in the increasingly sophisticated use of synthetic biology to engineer complex metabolic pathways into microbes ^8^.

It seems that the riddle of natural products is being gradually solved, as targeted, complex natural products can be obtained from engineered microbial hosts. However, when we examine these cases, it is obvious that the identification and characterization of key enzymes involved in the metabolic pathways of natural products might ultimately promote tremendous innovation and creativity. We can still use the story of artemisinin as an example. More than a decade ago, the amorpha-4,11-diene synthase (ADS) gene was cloned and identified in *A. annua* ^9^. Afterward, Martin *et al.* introduced this gene into *Escherichia coli*, which initially endowed the bacteria with the ability to produce amorphadiene, the sesquiterpene olefin precursor to artemisinin ^10^. Subsequently, by applying amorphadiene synthase and a novel cytochrome P450 monooxygenase (CYP71AV1) from *A. annua*, artemisinic acid can be efficiently produced from amorpha-4,11-diene in engineered *S. cerevisiae* via a three-step oxidation process ^11, 12^. Finally, Paddon *et al.* constructed the complete biosynthetic pathway of artemisinic acid by using *A. annua* artemisinic aldehyde dehydrogenase (ALDH1) and cytochrome CYB5, which provide a highly efficient biosynthetic route for artemisinic acid production, as the artemisinic acid titers can reach 25 g/L ^4^.

Consequently, step-by-step pathway exploration and engineering led to the discovery and application of new enzymes. Therefore, the characterization and elucidation of enzymes in natural product biosynthesis have become fundamental in the development of new drugs and the heterologous biosynthesis of active natural products. Here, we briefly review progress in the elucidation of enzymatic function in natural product biosynthesis over the past year.

## Identification of the enzymes DRS and DRR remove the last stumbling block in the complete synthesis of opioids

**Figure 1.**
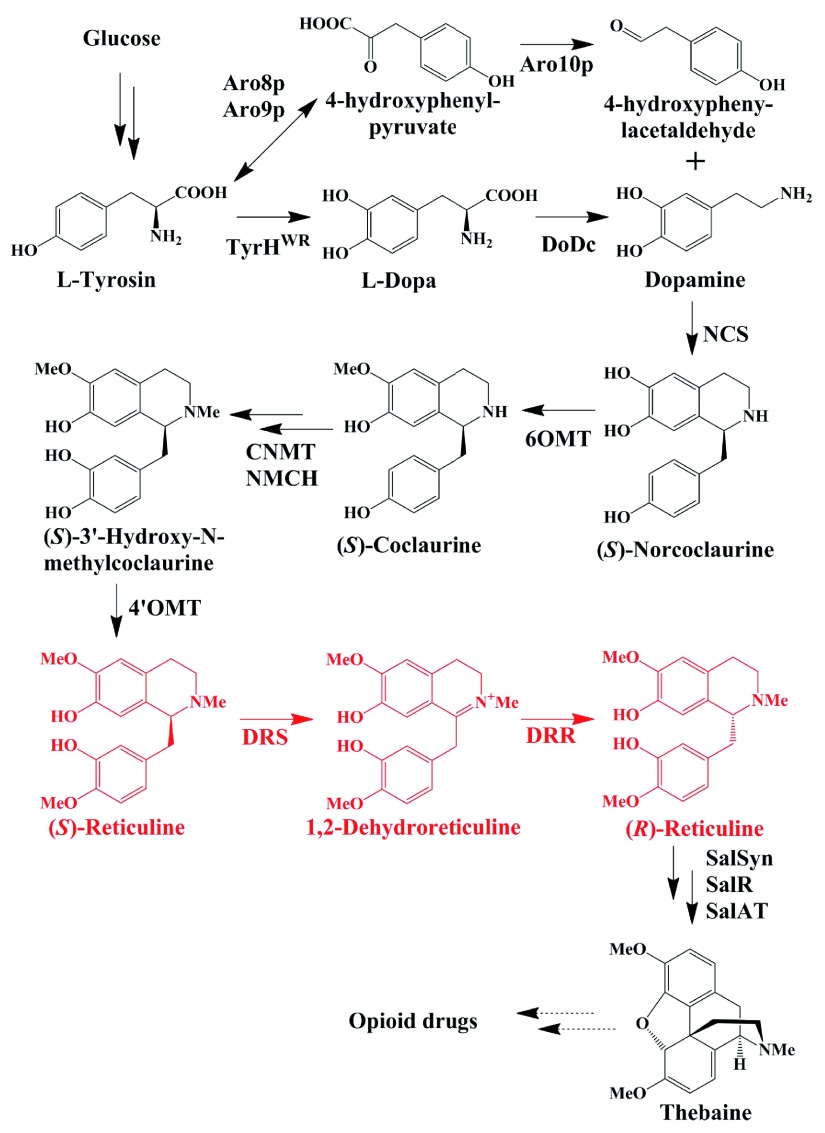
Biosynthetic scheme for production of opioids from sugar. DRR, 1,2-dehydroreticuline reductase; DRS, 1,2-dehydroreticuline synthase.

The secondary metabolites of higher plants include diverse chemicals, such as isoprenoids, phenolic compounds, and alkaloids. Among these natural products, alkaloids are very important medicines. Benzylisoquinoline alkaloids (BIAs) constitute a large and structurally diverse family of pharmaceutical alkaloids that contains 2,500 defined structures. BIAs, such as the well-known analgesic compounds morphine and codeine, and the antibacterial agents berberine, palmatine, and magnoflorine, are synthesized from tyrosine by members of the *Papaveraceae* and *Berberidaceae* plant families, as well as many others ^13^. Among these compounds, opioids are the primary drugs used for pain management and palliative care. Natural opiates, such as codeine and morphine, and semi-synthetic opioids, such as hydrocodone, hydromorphone, and oxycodone, are derived from the opium poppy ( *Papaver somniferum*). However, as the sole commercial source for pharmaceutical opiate production, industrial poppy farming is susceptible to environmental factors. Moreover, the chemical synthesis of these complex compounds is uneconomical because of their complex structures.

(S)-reticuline ( Figure 1) is the major branch-point intermediate in the biosynthesis of BIAs. However, only (R)-isomer of reticuline can be converted to opioids. Before the discovery of the enzyme that has the ability to convert (S)-reticuline to (R)-reticuline, many efforts over the past decade were made to engineer microbes to produce BIAs downstream of (S)-reticuline. By combining microbial and plant enzymes, Minami *et al.* successfully synthesized (S)-reticuline and its downstream products, magnoflorine and scoulerine, from dopamine in *E. coli* ^14^. In the same year, Hawkins and Smolke reported that by feeding chemically synthesized (R,S)-norlaudanosoline as a starting substrate, (R,S)-reticuline could be produced in engineered yeast, and that by using the human P450 enzyme CYP2D6 in this pathway, (R)-reticuline can be specifically converted to the morphinan alkaloid salutaridine ^15^. As noted in these studies, the starting substrates dopamine and (R, S)-norlaudanosoline are intermediate products in the BIA metabolic pathway. Years ago, Nakagawa *et al.* developed an *E. coli* fermentation system that yields (S)-reticuline from simple carbon sources ^16^. Very recently, DeLoache *et al.* demonstrated the production of the key BIA intermediate (S)-reticuline from glucose in *S. cerevisiae* ^17^. Thus, in microbes, the major branch-point intermediate (S)-reticuline in the BIA biosynthetic pathway can be efficiently synthesized from a simple carbon source, such as glucose.

Additionally, many studies have focused on the downstream (R)-reticuline metabolic pathway. In the opium poppy, the opioid compound thebaine can be converted to codeine, morphine, and so on, and can be produced by the N-demethylation of (R)-reticuline. Moreover, Thodey *et al.* recently reported that by engineering yeast to express heterologous genes from the plant *P. somniferum* and the bacterium *Pseudomonas putida* M10, the starting substrate thebaine can be converted to several types of opioids, such as codeine, morphine, hydromorphone, hydrocodone, and oxycodone ^18^.

It is obvious that the last stumbling block in the total synthesis of opioids in microbes is the gap between (S)-reticuline and (R)-reticuline. A few months ago in a study in *Science*, a metabolite analysis of mutant opium poppy plants and heterologous protein expression experiments demonstrated that the P450 module is responsible for the conversion of (S)-reticuline to 1,2-dehydroreticuline, but that the oxidoreductase module converts 1,2-dehydroreticuline to (R)-reticuline rather than functioning as a P450 redox partner ^19^. Subsequently, by using a plant transcriptome database and cloned genes from *P. somniferum*, another group identified candidate enzymes 1,2-dehydroreticuline synthase (DRS) and 1,2-dehydroreticuline reductase (DRR), which convert (S)-reticuline to 1,2-dehydroreticuline and then to (R)-reticuline ^20^. The identification of DRS and DRR theoretically paves the way for the total biosynthesis of opioids in microbes. Several weeks ago, the total biosynthesis of opioids in yeast was completed by Smolke’s group ^7^. In this landmark work, more than 20 enzymes derived from rodent, plants, bacteria, and yeast sources, were successfully expressed in engineered yeast ( Figure 1), which ultimately realized the complete biosynthesis of opioids from sugar ^7^. The success of microbial total biosynthesis of opioids reveals the significant functions of enzymes in recent drug development applications, as well as in the field of synthetic biology.

## Structural elucidation of key biosynthases facilitates drug development by combinatorial biosynthesis

The polyketides constitute a broad class of structurally complex, bioactive natural products that have many important therapeutic applications, such as antibiotics, anti-fungals, anti-parasitics, anticancer agents, and immunosuppressants. However, the emergence and spread of antibiotic resistance are gradually becoming global public health problems, which have intensified the efforts toward new drug discovery and combinatorial biosyntheses of bioactive molecules to broaden the spectrum of antibacterial agents ^21^.

The carbon framework of all polyketides is assembled by a polyketide synthase (PKS). Type I PKS modules act successively in polyketide chain elongation, processing, and termination. Each module contains acyl carrier protein (ACP), ketosynthase (KS), and acyltransferase (AT) domains that extend the linear sequence of an intermediate by two carbon atoms. The AT domain loads the ACP with a building block from a specific substrate, acylcoenzyme A (acyl-CoA), and the KS domain catalyzes C–C bond formation between the intermediate from the upstream module and the acyl-ACP. In addition, modules may contain domains that have the ability to modify the β-keto group to a hydroxyl group, a double bond, or a single bond, in the presence of ketoreductase, dehydratase, and enoylreductase domains, respectively. Therefore, the modular organization of these enzyme assembly lines holds tremendous promise for applications in synthetic biology and bioengineering ^22, 23^. By engineering an appropriately modified PKS, new polyketides can be rationally designed and produced ^24^. However, because the bacterial PKS module architecture and its conformational dynamics during polyketide chain elongation and processing are not yet well understood, the combinatorial biosynthesis of PKS often has been beset by many problems, such as inefficiency and dysfunction ^25^.

**Figure 2.**
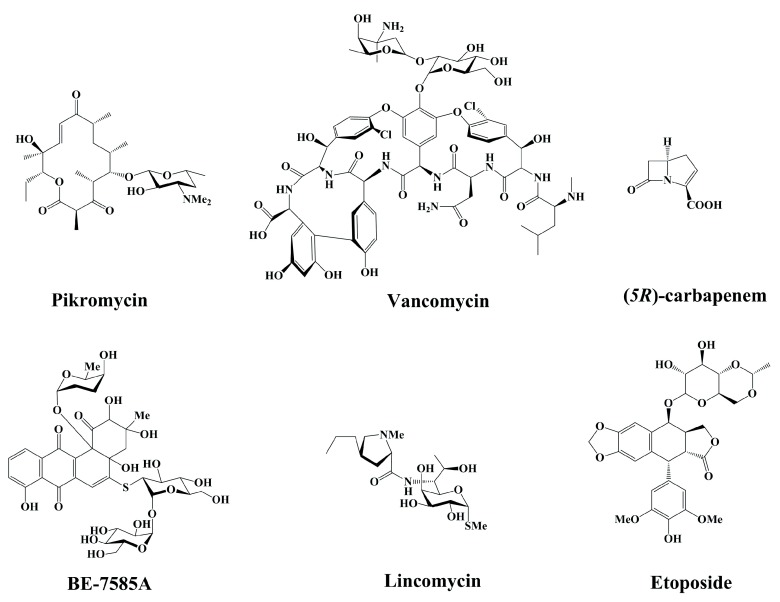
Chemical structure of pikromycin, vancomycin, carbapenem, BE-7585A, lincomycin, and etoposide.

Thus, for more than 10 years, enormous research efforts have focused on the structural biology of PKS, and consequently crystal structures have been reported for various excised PKS domains, KS-AT didomains, and docking domains ^26– 36^. In 2008, Maier *et al.* reported the crystal structure of porcine fatty acid synthase (FAS) and proposed a catalytic model of PKS ^37^. Although modular PKSs are thought to share a common ancestor with mammalian FAS ^38^, there are several differences between PKS and FAS. Recently, high-resolution information on the overall structure and organization of a complete PKS module was reported ^39, 40^. In this study, the crystal structure of a full-length PKS module from the pikromycin ( Figure 2) pathway of *Streptomyces venezuelae* was determined by electron cryomicroscopy. The structural data revealed the dynamics of ACP during sequential interactions with catalytic domains within the reaction chamber, and when transferring the elongated and processed polyketide substrate to the next module in the PKS pathway. The ACP domain optimally localizes its cargo for the next reaction. This series of structures provided significant new information about the dynamics of ACP and its interaction with the catalytic domains, as well as a new reference for future analyses of substrate selectivity and catalytic efficiency ^41^. The detailed insights of the PKS assembly line revealed in this study provide a new structural framework for the development of an effective approach for combinatorial biosynthesis.

In addition to PKS, non-ribosomal peptide synthetases (NRPSs) and PKS-NRPS hybrids represent a large multi-enzyme family that is involved in the biosynthesis of many microbial peptides and glycopeptide antibiotics, such as teicoplanin and vancomycin ( Figure 2). *Streptomyces* spp. produce natural compounds, often with highly complex structures, which are difficult to produce or are not economically feasible to produce by chemical synthesis. Therefore, understanding biosynthetic pathways is of great clinical importance, and it is essential for combinatorial biosynthesis. Several months ago, Haslinger *et al.* reported the structure of a previously uncharacterized “X-domain”, which is strictly conserved in the final module of all glycopeptide NRPSs ^42^. The X-domain interacts with two cytochrome P450 molecules, and the structures of the X-domain, both in isolation and in complex with the first P450 oxygenase protein involved in teicoplanin biosynthesis, revealed the inactive nature of the X-domain, as well as how oxygenase recruitment occurs.

β-lactam compounds account for more than half of the global antibiotic drug market ^43^. However, the increased incidence of bacterial β-lactam resistance has led to an increasing reliance on a relatively new subclass of these drugs, which are known as carbapenems ( Figure 2) ^43, 44^. Therefore, a better understanding of the biosynthetic mechanisms of carbapenems could facilitate further carbapenem-derived drug development to combat resistance. During the biosynthesis of these compounds, it is unclear whether carbapenem synthase (CarC) catalyzes the inversion of C5 in the β-lactam ring. By combining x-ray crystallography with multiple spectroscopic probes, Chang *et al.* recently revealed the mechanism by which the CarC enzyme inverts the precursor configuration of C5 to that of its mirror image ^45^. A greater understanding of the structure and function of CarC is expected to aid in the efforts to bioengineer new carbapenems.

## The secret of sulfur in natural products has been revealed

Many natural products contain sulfur, which is a ubiquitous element that is essential for nutrition and metabolism in living systems. However, the mechanism by which this atom is incorporated into sulfur-containing compounds remains poorly understood. BE-7585A ( Figure 2) is a 2-thiosugar-containing antibiotic that is produced by *Amycolatopsis orientalis* ^46^. To study the production of the 2-thiosugar moiety in BE-7585A, Sasaki *et al.* identified a thiazole synthase homologue, BexX, which is responsible for the biosynthesis of 2-thiosugars in BE-7585A ^47^. Briefly, as a 2-thioglucose synthase, BexX converts glucose-6-phosphate to 2-thioglucose. However, sulfur-carrier proteins, the donors of the sulfur moiety, are also involved in the biosynthesis of cysteine and molybdopterin, which are primary metabolites. In this case, sulfur transfer proteins from primary metabolic pathways are hijacked to facilitate the biosynthesis of secondary metabolites, which represents a rare link between primary and secondary metabolism.

Another study investigated the sulfur-containing compound lincomycin A ( Figure 2), an efficient antibacterial that is produced by *Streptomyces lincolnensis* and used for agricultural purposes ^48^. As is known, mycothiol (MSH) is a cysteinyl pseudo-disaccharide that is found in Gram-positive actinobacteria, and the conjugation of this thiol-containing compound to electrophilic toxins facilitates their excretion from the bacterial cell ^49^. Ergothioneine (EGT), a histidine betaine derivative, is another thiol that is produced by actinomycetes ^50^. In the biosynthesis of lincomycin A, both MSH and EGT play important roles in the incorporation of sulfur. EGT acts as a carrier in the template-guided molecular assembly, and MSH is the sulfur-donor for lincomycin maturation after thiol exchange ^51^. In the biosynthesis of lincomycin A, these thiols function through two unusual S-glycosylations that program lincosamide transfer, activation, and modification, providing the first paradigm for EGT-associated biochemical processes and the poorly understood MSH-dependent biotransformations ^51^. This study also demonstrated that the integration of primary metabolites (such as MSH and EGT) and secondary metabolites is crucial for the biosynthesis of complex molecules ^52^.

## Etoposide: the next candidate for total biosynthesis in microbes?

As previously mentioned, numerous clinically used drugs, such as artemisinin and opioids, are derived from plant secondary metabolites. However, little is known about their biosynthetic genes, and this prevents highly efficient production of these compounds by engineered hosts. Since very few complete pathways of plant-derived drugs have been identified, heterologous production has been successfully applied for a few plant-derived drugs.

Months ago, the biosynthetic pathway of another efficient anticancer drug, etoposide ( Figure 2), was identified ^53^. As is known, etoposide is very difficult to obtain because its precursor is derived from the very slow-growing mayapple plant ( *Podophyllum peltatum*), which is also an endangered species ^54^. Therefore, Lau and Sattely worked out the pathway that produces the precursor of etoposide in the mayapple plant using bioinformatics, heterologous enzyme expression, and kinetic characterization ^53^. Notably, only six enzymes are responsible for the complete biosynthesis of the etoposide aglycone by the mayapple plant. By transferring these six genes to the tobacco plant, the etoposide aglycone was successfully produced in a heterologous host.

## Perspectives

**Figure 3.**
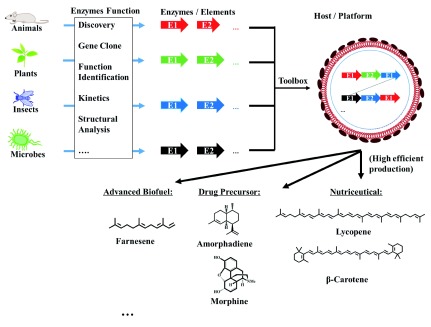
Synthetic biology: enzyme functions for natural product biosynthesis.

By reviewing the literature and some recent remarkable case studies of natural product biosyntheses or enzymatic functions, the significance and utility of enzymatic functions for synthetic biology applications can be demonstrated. As Charles Dickens said at the beginning of *A Tale of Two Cities*, “It was the best of times, it was the worst of times”, but now we have to admit that this is the best of times for synthetic biology. Thanks to the advent and widespread application of “-omics” techniques as well as computational biology techniques, more and more enzymes involved in natural product biosyntheses have been revealed. In addition, with the rapid emergence of new techniques, such as the latest powerful gene-editing tool CRISPR/Cas9, the roles of targeted genes that encode enzymes involved in natural product biosyntheses can also be efficiently identified. Finally, by using electron cryomicroscopy, structural biologists are now able to solve the complex catalytic mechanisms of enzymes. In an era of exponentially accumulating data, any optimization or innovation of a new technique could speed up the exploration of enzymatic functions. Consequently, more BioBricks or components are increasingly being deposited into the synthetic biology toolbox ( Figure 3), which can be used in drug development and combinatorial biosynthesis by designing new routes, and merging biosynthetic and synthetic routes.
